# Daily AI-Based Treatment Adaptation under Weekly Offline MR Guidance in Chemoradiotherapy for Cervical Cancer 1: The AIM-C1 Trial

**DOI:** 10.3390/jcm13040957

**Published:** 2024-02-07

**Authors:** Fabian Weykamp, Eva Meixner, Nathalie Arians, Philipp Hoegen-Saßmannshausen, Ji-Young Kim, Bouchra Tawk, Maximilian Knoll, Peter Huber, Laila König, Anja Sander, Theresa Mokry, Clara Meinzer, Heinz-Peter Schlemmer, Oliver Jäkel, Jürgen Debus, Juliane Hörner-Rieber

**Affiliations:** 1 Department of Radiation Oncology, Heidelberg University Hospital, 69120 Heidelberg, Germanyjuliane.hoerner-rieber@med.uni-heidelberg.de (J.H.-R.); 2 Heidelberg Institute of Radiation Oncology (HIRO), 69120 Heidelberg, Germany; 3 National Center for Tumor Diseases (NCT), 69120 Heidelberg, Germany; 4 Clinical Cooperation Unit Radiation Oncology, German Cancer Research Center (DKFZ), 69120 Heidelberg, Germany; 5 Division of Molecular and Translational Radiation Oncology, German Cancer Research Center (DKFZ), 69120 Heidelberg, Germany; 6 Clinical Cooperation Unit Molecular Radiation Oncology, German Cancer Research Center (DKFZ), 69120 Heidelberg, Germany; 7 Institute of Medical Biometry, University of Heidelberg, 69120 Heidelberg, Germany; 8 Department of Radiology, Heidelberg University Hospital, 69120 Heidelberg, Germany; 9 Department of Radiology, German Cancer Research Center (DKFZ), 69120 Heidelberg, Germany; 10 Division of Medical Physics in Radiation Oncology, German Cancer Research Center (DKFZ), 69120 Heidelberg, Germany; 11 Heidelberg Ion-Beam Therapy Center (HIT), Department of Radiation Oncology, Heidelberg University Hospital, 69120 Heidelberg, Germany; 12 German Cancer Consortium (DKTK), Partner Site, 69120 Heidelberg, Germany

**Keywords:** online adaptive radiotherapy, MR guidance, cervical cancer, chemoradiotherapy

## Abstract

(1) **Background:** External beam radiotherapy (EBRT) and concurrent chemotherapy, followed by brachytherapy (BT), offer a standard of care for patients with locally advanced cervical carcinoma. Conventionally, large safety margins are required to compensate for organ movement, potentially increasing toxicity. Lately, daily high-quality cone beam CT (CBCT)-guided adaptive radiotherapy, aided by artificial intelligence (AI), became clinically available. Thus, online treatment plans can be adapted to the current position of the tumor and the adjacent organs at risk (OAR), while the patient is lying on the treatment couch. We sought to evaluate the potential of this new technology, including a weekly shuttle-based 3T-MRI scan in various treatment positions for tumor evaluation and for decreasing treatment-related side effects. (2) **Methods**: This is a prospective one-armed phase-II trial consisting of 40 patients with cervical carcinoma (FIGO IB-IIIC1) with an age ≥ 18 years and a Karnofsky performance score ≥ 70%. EBRT (45–50.4 Gy in 25–28 fractions with 55.0–58.8 Gy simultaneous integrated boosts to lymph node metastases) will be accompanied by weekly shuttle-based MRIs. Concurrent platinum-based chemotherapy will be given, followed by 28 Gy of BT (four fractions). The primary endpoint will be the occurrence of overall early bowel and bladder toxicity CTCAE grade 2 or higher (CTCAE v5.0). Secondary outcomes include clinical feasibility, quality of life, and imaging-based response assessment.

## 1. Introduction

Cervical cancer is one of the most common cancer types in women worldwide [1]. Definitive concurrent chemoradiotherapy (CCRT), followed by brachytherapy, is a standard of care for patients with locally advanced cervical cancer [2]. However, some patients develop high-grade toxicity. The EMBRACE study, one of the largest studies on CCRT and brachytherapy in cervical cancer, reported an actuarial 5-year cumulative incidence of grade 3 or higher in the bladder and a bowel toxicity of 15.3% [3]. It is well known that gastrointestinal (GI) and bladder toxicity impair quality of life [4]. Bowel and bladder toxicity are known to have the highest negative impact on patient-reported outcomes [5]. Up to 10% of patients who develop acute diarrhea due to pelvic irradiation have persistent symptoms for more than five years [6]. Pelvic radiation disease is a lately branded term for persisting radiation-induced symptoms and is estimated to be nearly twice as common as Crohn’s disease, which in turn is one of the most common inflammatory bowel diseases [7].

The implementation of modern conformal radiotherapy delivery methods, like intensity-modulated therapy (IMRT), has led to decreased irradiation volumes and reduced high-grade (G3/4) toxicity over the last few years [8,9]. Consequently, IMRT enables improved GI and bladder-related patient-reported symptoms [10]. Nonetheless, GI and bladder toxicity is still high [11]. Target volume delineation for cervical cancer comprises the cervix and the uterus, which are known to show significant motion, mostly due to the close proximity to hollow organs at risk (OARs) [11,12]. Therefore, in conventional CCRT using IMRT, large safety margins still have to be applied and should routinely include much of the surrounding healthy tissue (e.g., bowel and bladder), potentially increasing treatment-related side effects. Furthermore, daily image guidance to account for positional changes is conventionally performed with low-dose cone beam computer tomography (CBCT) scans which only offer low soft-tissue contrast and hence no direct visualization of the tumor and the surrounding organs at risk (OAR).

Lately, the radiotherapy device ETHOS (VARIAN; Palo Alto, CA, USA), which was recently installed at the Clinical Cooperation Unit Radiation Oncology at the German Cancer Research Center, is the first commercially available linear accelerator which offers daily high-quality CBCT. Therefore, the treatment plan can be adapted daily to the changes in anatomy (adaptive treatment) while the patient is lying on the treatment couch [13]. This enables a reduction in safety margins and yields the possibility of reducing toxicity; however, it prolongs treatment sessions [14]. To limit time expenses, online adaptation at the ETHOS is artificial intelligence (AI)-based, offering an automatically generated and daily adapted treatment plan. 

Magnetic resonance imaging (MRI) scans have a high soft-tissue contrast and are therefore used for initial tumor staging and cervical cancer follow-up [15]. Based on the increasingly successful utilization of MR guidance in BT planning, it has already been desired for image guidance in EBRT [11,16]. Nonetheless, due to its broad availability and practical reasons, CBCT scans are used instead of MR scans as image guidance for EBRT. However, as stated above, conventional CBCT scans only have a low soft-tissue contrast and do not allow for the exact localization of OAR or macroscopic tumor volume. 

The German Cancer Research Center (DKFZ) is one of the few facilities worldwide that combines the online adaptive radiotherapy device ETHOS with one of the latest imaging modalities, the 3T MRI scanner VIDA (Siemens, Germany). Patients can be shuttled in treatment positions from the MRI scanner to the ETHOS once a week, allowing for offline MR guidance on a regular basis. Besides daily AI-supported CBCT treatment adaptation, this offers the unique potential to utilize high-resolution functional MR imaging in treatment positions for additional plan adaptation and early response assessment. 

## 2. Methods

### 2.1. Study Design

The trial will be performed as a prospective single-arm phase-II proof-of-concept study.

The planned recruitment time for a total of 40 patients is 24 months. The inclusion and exclusion criteria are described in Table 1 and Table 2.

EBRT will be planned based on pre-therapeutic MRI and planning CT, and will consist of 25–28 fractions of 1.8 Gy leading to a total dose of 45–50.4 Gy using intensity-modulated radiotherapy (IMRT). A simultaneous integrated boost of 2.1–2.2 Gy will be applied to eventual lymphatic node metastases, leading to a boost plan dose of 55.0–58.8 Gy. Concurrent platinum-based chemotherapy (e.g., cisplatin 40 mg/m^2^ weekly; aim: 5–6 cycles) will be applied during EBRT according to current international and German guidelines [17]. Target volume delineation for EBRT was adapted from Lee et al. [18], as well as from the consensus guidelines of the Radiation Therapy Oncology Group [19]. Furthermore, an adapted version of the recently introduced internal target volume (ITV) concept of the EMBRACE-II study will be used to account for intrafractional organ motion [20].

- Gross tumor volume (GTV): macroscopic contrast-enhanced tumor in the planning MR and, if definable, in the planning CT.
- Clinical target volume (CTV):
  ■ CTV 1: GTV, cervix, and uterus.
  ■ CTV 2: paravaginal/parametrial tissue; proximal half of the vagina (proximal two-thirds if infiltrated).
  ■ CTV 3: internal/external/common iliacal, as well as presacralic (S 1/2), lymphatic drainage area (including surgical clips and lymphoceles); cranial border: aortic bifurcation; caudal border: caput femoris (external iliacal) or cranial vaginal end (internal iliacal).
- Depending on the planning CT and MRI scan, CTV 1 and CTV 2 will be combined and adapted to form an ITV, which accounts for the organ motion during the adaptation process.
- A margin of 5 mm will be added to the ITV and CTV 3 to generate the planning target volume (PTV).

All patients will receive a pre-EBRT and (during EBRT) a pre-BT pelvic MRI scan for diagnostic and treatment planning purposes (contrast-enhanced), as well as once weekly during CCRT for image guidance (non-contrast-enhanced) in treatment positions. Patients will be immobilized and shuttled (Symphony™ Patient Transport System) from the 3T MRI scanner (VIDA, Siemens) to the ETHOS system, which is located in the same vault. The shuttle system is MR-compatible and utilizes low-friction air bearing to transition patients from one modality to another, eliminating the need to manually lift the patient. MR coil fixation systems from QFix allow for the alignment of MR coils without touching or deforming the patient’s surface. Furthermore, MRI will be regularly performed during follow-up visits (contrast-enhanced).

**Figure 1:**
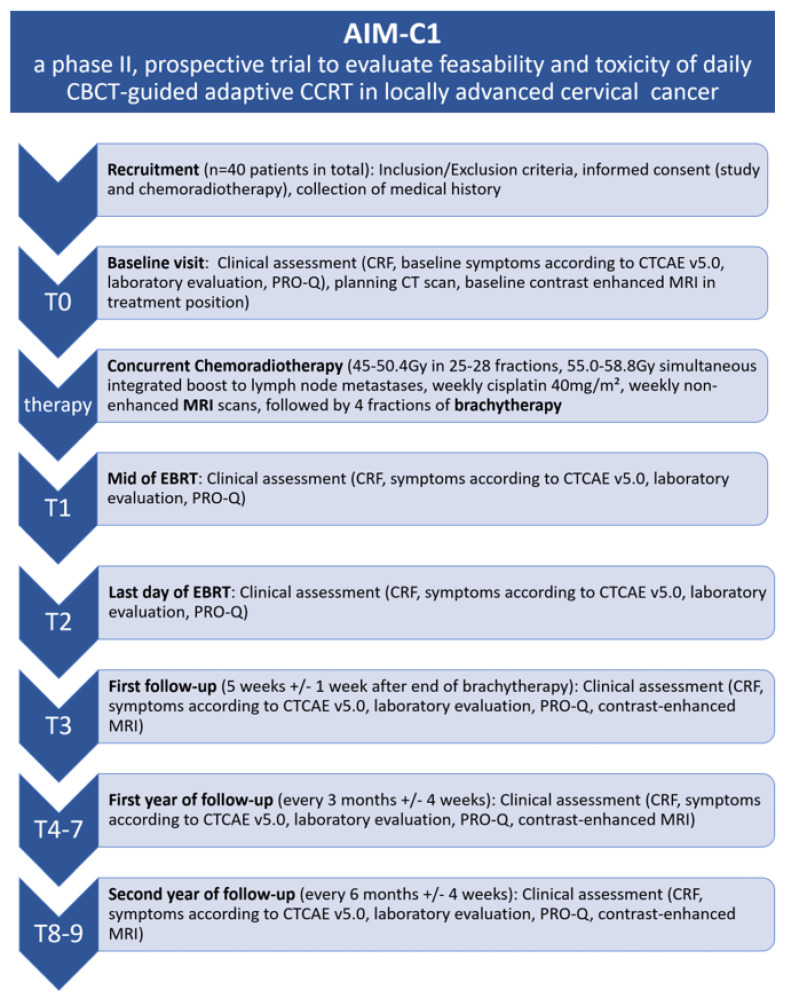
Flowchart of the AIM-C1 study.

Patients will be scheduled for study visits in the middle of CCRT and at the end of CCRT. As per institutional clinical standards, patients receive their first follow-up study visit five weeks after the end of brachytherapy, which functions as a baseline for subsequent visits every three months after the end of radiotherapy and then every three to six months up to two years of follow-up. Follow-up visits include a clinical assessment, as well as a contrast-enhanced MRI, of the pelvis. The respective study visits also include an analysis of quality of life (using EORTC QLQ C-30 and EORTC QLQ CX-24 questionnaires). Baseline symptoms and toxicities will be assessed according to CTCAE V5.0. The flowchart of the AIM-C1 study is found in Figure 1. 

### 2.2. Investigated Populations

The intention-to-treat population (ITT) comprises all enrolled patients who fulfilled the in-/exclusion criteria and are randomized in the trial. The patients will be analyzed irrespective of the treatment actually received. This will be the primary population for evaluating all efficacy endpoints and subject characteristics.

The per protocol (PP) population will represent the secondary efficacy analysis population (for sensitivity analysis purposes) and will comprise all patients of the ITT population, if the following criteria are additionally met:

- All of the inclusion criteria (none of the exclusion criteria are fulfilled);
- Treatment, as outlined in the protocol, without major protocol violations.

### 2.3. Sample Size Calculation

This is an exploratory trial which aims to provide a first reliable rate estimate of the primary endpoint, serving as a starting point for subsequent potential randomized trials, defined as the occurrence of treatment-related bowel or bladder CTCAE V5.0 toxicity (G2 or higher) assessed at the first 3 months after the beginning of treatment. 

Bladder toxicity of grade 2 (G2) or higher (frequency/urgency, incontinence, spasm, stenosis, cystitis, bleeding, fistula, etc.) was described to be 6.1% after three months since the beginning of the CCRT for cervical carcinoma in the EMBRACE study [21]. Overall, bowel morbidity of G2 or higher (diarrhea, flatulence, incontinence, stenosis/stricture fistula, etc.) was reported to be approximately 4% at three months [22]. Thus, the G2 or higher bowel and bladder toxicity is approximately 10% with the conventional irradiation technique.

With a sample size of *n* = 40 patients, and assuming that a G2 or higher toxicity rate of 8% can be achieved through daily adaptation and weekly MRI guidance, the 90% Wilson score rate confidence interval will be [3.3%, 18.0%] with a width of 14.7 percent points, thus illustrating the evidence that can be gained with our trial. Our primary goal is to provide a first estimate of the toxicity rate for experimental treatment. Sample size calculation was carried out using PASS v16.0.3. 

### 2.4. Study Objectives

The purpose of this phase-II proof-of-concept study is to reduce irradiation-induced toxicity and morbidity in cervical cancer patients through daily CBCT-based and AI-supported adaptation under weekly MR guidance. External beam radiotherapy itself, concomitant chemotherapy, and the subsequent brachytherapy are performed according to national guidelines and internal standard-operating procedures (SOPs) and are therefore clinical routine.

The primary endpoint is defined as the occurrence of treatment-related bowel or bladder CTCAE V5.0 toxicity (G2 or higher) within the first 3 months. 

The secondary objectives include an evaluation of treatment efficacy and patient survival, as well as changes in quality of life. This also includes local and distant tumor control, according to RECIST v1.1, as well as progression-free survival and overall survival. Furthermore, the clinical feasibility of MR guidance, especially plan adaptation using the AI-based ETHOS system, will be studied. In addition, morphological, functional, and motional changes in MRI will be compared to those in CT scans. Finally, secondary objectives encompass a collection of treatment plans and irradiation parameters, as well as imaging data and quality assurance results for further analysis and the planning of follow-up projects, including how often the adapted plan was chosen, the dosimetric benefits of adaptation, dosimetric comparison with other radiotherapy techniques, the analysis of plan robustness, the evaluation of AI-based adaptation, the analysis of image registration algorithms, as well as the assessment of fraction dose accumulation methods.

### 2.5. Withdrawal of Patients

A subject may voluntarily discontinue participation in this study at any time at their own request. In addition, study treatment will be discontinued if unmanageable toxicity is documented or if the principal investigator decides to terminate the study. Moreover, a subject will be withdrawn from the protocol if, in the investigator’s opinion, continuation of the trial would be detrimental to the subject’s well-being. If the subject withdraws from the trial, no further evaluations will be performed and no additional data will be collected. The principal investigator may ask for the patient’s consent in order to continue using any data collected before such withdrawal from the trial. Without this consent, all collected patient data must be deleted completely from the study database.

In all cases, the reason for withdrawal must be recorded in the case report form and in the subject’s medical records. In the case a subject withdrawing at their own request, the reason should be disclosed and documented. All efforts will be made to follow up with the subject and all examinations scheduled for the final trial day will be performed and documented as much as possible. All ongoing adverse events (AEs) of withdrawn patients have to be followed up until no more signs and symptoms are verifiable or the subject is in stable condition.

### 2.6. Prior and Concomitant Illnesses and Treatments

Relevant additional illnesses present at the time of informed consent are regarded as concomitant illnesses and will be documented in the patient chart. Abnormalities which appear for the first time or worsen (intensity and frequency) during the trial are adverse events (AEs) and must be documented on the appropriate pages of the case report form (CRF). Relevant additional treatments administered to the patients on entry to the trial or at any time during the trial are regarded as concomitant treatments and must be documented on the appropriate pages of the CRF. During radiation therapy, medication required for concomitant illnesses (i.e., hypertension, thyroid disease, hyperlipidemia, etc.) should be applied. Concomitant medication should be discussed with the principal investigator on an individual basis. The concomitant systemic therapy, as indicated by the national guidelines, represents a standard of care and is not a part of the study protocol. Additional concomitant systemic therapy or other anti-tumor medication are also not part of the study protocol and are only allowed if in accordance with the treating radiation oncologist.

### 2.7. Statistical Analysis

The primary endpoint will be analyzed in the safety population. The toxicity rate will be calculated using a 90% Wilson-type confidence interval. No imputation of the primary endpoint will be conducted. Further analyses of the primary endpoint will be carried out in the ITT and PP population.

Analyses of secondary endpoints will be descriptive and will include the calculation of appropriate summary measures of the empirical distributions (continuous variables: mean, standard deviation, median, interquartile range, minimum, and maximum; categorical variables: frequencies and percentages). 

Time-to-event endpoints will be visualized by means of Kaplan–Meier plots and/or cumulative incidence functions. Secondary efficacy endpoints will be assessed in the ITT population, with further analyses conducted for the PP population. 

The method of Kaplan and Meier will be used to estimate the distributions of PFS and OS. Additionally, 95% log–log-type confidence intervals will be given for the Kaplan–Meier estimates.

Distant and regional tumor control will be assessed via cumulative incidence functions, taking the competing event death into account. The 95% confidence intervals for the cumulative incidence will be given.

QoL, as well as tumor size and other MRI-based continuous parameters, will be summarized by the mean, standard deviation, median, minimum, and maximum, and will be plotted based on time. The change from baseline over time will be examined descriptively. The safety analysis will comprise a tabulation and summary of adverse and serious adverse events and will be based on the safety population. The relative frequency of adverse and serious adverse events are summarized along with two-sided confidence intervals with a Wilson score of 95%.

### 2.8. Ethical Aspects

This study is carried out in accordance with the Declaration of Helsinki and received approval by the Ethics Committee of the Medical Faculty of the University of Heidelberg (S-466/2023 date of approval: 27 September 2023).

## 3. Discussion

CCRT is a well-established curative treatment concept for locally advanced cervical cancer patients, albeit the irradiation dose is conventionally barely adjusted to the individual patient [2]. Nearby pelvic OAR (like the bowel and the bladder, as well as the uterus itself) are known to show high-position variability over time [11,12]. To account for this variability, when using conventional techniques, large safety margins need to be applied, leading to an irradiation dose exposition of surrounding radiosensitive organs at risk (OARs), and hence toxicity and morbidity [5]. Bowel and bladder morbidity after pelvic irradiation often persist for years, have a dramatically negative impact on quality of life, and even become further important in times of increasing long-term cancer survivorship [4,6,23].

The ETHOS radiotherapy device offers an AI-supported daily treatment plan adaptation and thereby reduces the amount of unnecessary irradiated healthy tissue [24]. 

The first results in cervical cancer patients demonstrated the feasibility and dosimteric benefits of the ETHOS radiotherapy system [25,26,27,28]. However, these studies were partly from in silico trials, and clinical results are still missing. 

As one of the few centers worldwide, we can combine the latest radiotherapy device with weekly offline MR-based adaptation in treatment positions, including functional imaging. MR-guided radiotherapy with its superior soft-tissue contrast is believed to facilitate the precise detection of the current tumor dimension. This is especially important for cervical cancer, which often shrinks by up to 60–80% over the radiotherapy period, underlining the potential for adaptive treatment strategies [29]. There is growing evidence for the utilization of functional MRI in gynaecological malignancies [30]. Early tissue changes, visualized by MRI scans during the chemoradiotherapy course, have the potential to predict tumor responses and late toxicity, and thus may be used to determine the optimal individual irradiation dose [31,32,33]. 

In summary, daily adaptive cone beam CT (CBCT)-guided radiotherapy at the ETHOS offers the opportunity to significantly optimize treatment strategies regarding target coverage and toxicity. The additional integration of weekly MRI in treatment positions potentially allows for the functional non-invasive assessment of tissue perfusion, hypoxia, or cellular density. These data could be used for treatment adaptation and the assessment of early tissue changes. Up to now, there are only few data about daily adaptive CCRT in locally advanced cervical cancer. The aim of the current trial is therefore to evaluate the potential of daily adaptive CCRT in cervical cancer and analyze the benefit of the additional integration of high-resolution MRI in treatment positions for toxicity and response assessment. Our study will serve as a starting point for a potential subsequent randomized trial.

## Figures and Tables

**Table 1:** Inclusion criteria of the AIM-C1 study.

Biopsy-proven cervical cancer, including squamous cell carcinoma, adenocarcinoma, or adeno-squamous cell carcinoma FIGO-stage IB/IIA (if medically inoperable or ≥3 risk factors *), IIB, IIIA, IIIB, and IIIC1 (staging: pelvic MRI and thoracoabdominal CT) Indication and eligibility for definitive chemoradiotherapy, including brachytherapy discussed in an interdisciplinary tumor board Karnofsky performance score ≥ 70% 18–80 years of age Capacity of the patient to consent to participation in the study Patient-informed consent

* risk factors: L1, V1, deep stromal invasion, tumor size ≥ 4 cm, G3.

**Table 2:** Exclusion criteria of the AIM-C1 study.

FIGO-Stage IIIC2 and IV
Paraaortic Lymphatic Node Metastases
Small-cell neuroendocrine cancer, melanoma, and other rare cancers of the cervix Previous radiotherapy of the pelvic region Previous total or partial hysterectomy Contraindications against performing contrast-enhanced MRI scans (pacemakers, other implants making MRI impossible, and allergies to gadolinium (GD)-based contrast agents) Claustrophobia Pregnant or lactating women Other primary malignancies within 5 years before, except carcinoma in situ of the cervix and basal cell carcinoma of the skin Patient is enrolled in another study, which could influence the outcome of the presented study

## Data Availability

The data presented in this study will be available on reasonable request from the corresponding author.
